# Boosting Charge Utilization in Self‐Powered Photodetector for Real‐Time High‐Throughput Ultraviolet Communication

**DOI:** 10.1002/advs.202301585

**Published:** 2023-06-04

**Authors:** Tian Ouyang, Xuan Zhao, Xiaochen Xun, Fangfang Gao, Bin Zhao, Shuxin Bi, Qi Li, Qingliang Liao, Yue Zhang

**Affiliations:** ^1^ Academy for Advanced Interdisciplinary Science and Technology Beijing Advanced Innovation Center for Materials Genome Engineering University of Science and Technology Beijing Beijing 100083 P. R. China; ^2^ Beijing Key Laboratory for Advanced Energy Materials and Technologies School of Materials Science and Engineering University of Science and Technology Beijing Beijing 100083 P. R. China

**Keywords:** charge utilization, self‐powered photodetectors, Ti_3_C_2_T*_x_*, ultraviolet communication, ZnO

## Abstract

Ultraviolet (UV) communication is a cutting‐edge technology in communication battlefields, and self‐powered photodetectors as their optical receivers hold great potential. However, suboptimal charge utilization has largely limited the further performance enhancement of self‐powered photodetectors for high‐throughput communication application. Herein, a self‐powered Ti_3_C_2_T*_x_*‐hybrid poly(3,4 ethylenedioxythiophene):poly‐styrene sulfonate (PEDOT:PSS)/ZnO (TPZ) photodetector is designed, which aims to boost charge utilization for desirable applications. The device takes advantage of photothermal effect to intensify pyro‐photoelectric effect as well as the increased conductivity of the PEDOT:PSS, which significantly facilitated charge separation, accelerated charge transport, and suppressed interface charge recombination. Consequently, the self‐powered TPZ photodetector exhibits superior comprehensive performance with high responsivity of 12.3 mA W^−1^ and fast response time of 62.2 µs, together with outstanding reversible and stable cyclic operation. Furthermore, the TPZ photodetector has been successfully applied in an integrated UV communication system as the self‐powered optical receiver capable of real‐time high‐throughput information transmission with ASCII code under 9600 baud rate. This work provides the design insight of highly performing self‐powered photodetectors to achieve high‐efficiency optical communication in the future.

## Graphical abstract

The photothermal effect intensifies the pyro‐photoelectric effect as well as the optimized carrier transport rate is applied to promote charge separation, transfer and suppress charge recombination to boost charge utilization in the self‐powered photodetector. Thereafter, the UV communication system based on the self‐powered photodetector presents real‐time high‐throughput information transmission under 9600 baud rate.

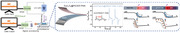

## 1 Introduction

Optical wireless communication (OWC) offers an unlicensed and secured bandwidth that has attracted considerable attention.^[^ ^1^ ^]^ Among them, ultraviolet (UV) communication technology is highly appealing owing to its intrinsic advantages of non‐line‐of sight and background noise negligibility.^[^ ^2^ ^]^ Photodetectors as optical receivers achieving high‐efficiency photoelectric conversion of signals are critical parts of UV communication systems. The conventional photomultiplier tube that is still being used has a large volume, high power consumption, and is fragile.^[^ ^3^ ^]^ Other group‐III‐nitride‐based photodetectors suffer from costly materials, cumbersome fabrication process, and tedious technical requirements.^[^ ^4^ ^]^ The existence of these limitations fails to meet the growing industrial requirements of UV communication technology in terms of cost‐saving, energy efficiency, and portability. In this regard, it is highly desired to develop efficient low cost and energy‐saving photodetectors for UV communication systems.

In the last decade, photovoltaic‐type self‐powered UV photodetectors without an external energy power source have been investigated intensively.^[^ ^5^ ^]^ In particular, the zinc oxide (ZnO)‐based self‐powered UV photodetector has developed vigorously owing to its wide bandgap, large exciton binding energy, and low cost.^[^ ^6^ ^]^ In order to move toward practical application, plenty of effort has been paid to pursue high‐performance self‐powered UV photodetectors.^[^ ^7^ ^]^ Generally, the built‐in electric field acts like a driving force that can efficiently separate photo‐generated electron‐hole pairs without external bias.^[^ ^8^ ^]^ Therefore, several strategies have been adopted to strengthen the built‐in electric field to further ameliorate performance of self‐powered photodetectors.^[^ ^9^ ^]^ Besides, combined with other intrinsic physical effects of materials is another powerful weapon to enhance the photovoltaic effect.^[^ ^10^ ^]^ Specifically, the pyro‐phototronic effect by naturally coupling the light‐induced pyroelectric effect and photovoltaic effect can enhance the performances of self‐powered photodetectors.^[^ ^11^ ^]^ Nonetheless, the space of the device performance improvement through the effect is also confined by the limited rate of temperature change. Thereby, various kinds of efforts have been devoted to further enhancing self‐powered photodetector performance based on the pyro‐phototronic effect.^[^ ^12^ ^]^ Recently, MXene as a new type of transition metal carbide/carbonitride, which shows metal‐like conductivity together with exhibits nearly 100% internal light‐to‐heat conversion efficiency.^[^ ^13^ ^]^ These special properties are potentially able to harmoniously exert synergistic effects in optimizing photodetector performances, which have not been explored. In addition, self‐powered photodetectors offer formidable evolution opportunities for UV communication technology and have garnered significant research interest. However, the state‐of‐art comprehensive performance of these photodetectors resulting from suboptimal charge utilization still cannot meet the demand for throughput communication practical applications.^[^ ^14^ ^]^

Herein, a self‐powered Ti_3_C_2_T*_x_*‐hybrid poly(3,4 ethylenedioxythiophene):poly‐styrene sulfonate (PEDOT:PSS)/ZnO (TPZ) photodetector is designed, which aims to boost charge utilization and enable superior photoresponse performance. The efficient photothermal effect intensifying pyro‐photoelectric effect coupled with increased conductivity of the PEDOT:PSS significantly facilitated charge separation, accelerated charge transport, and suppressed interface charge recombination without external bias. Taking full advantage of this strategy, the self‐powered TPZ photodetector exhibits desirable comprehensive performance with a high responsivity up to 12.3 mA W^−1^, fast response speed (62.2 µs) together with outstanding reversible and stable cyclic operation. Ultimately, we successfully demonstrated the use of the self‐powered TPZ photodetector as an optical receiver in the integrated UV communication system enabling high‐throughput transmitting texts at a 9600 baud rate. This work would provide guidelines for rational design of high‐performance self‐powered photodetectors towards efficient UV communication thus making it more perspective in practical application.

## 2 Results and Discussion

**Figure 1:**
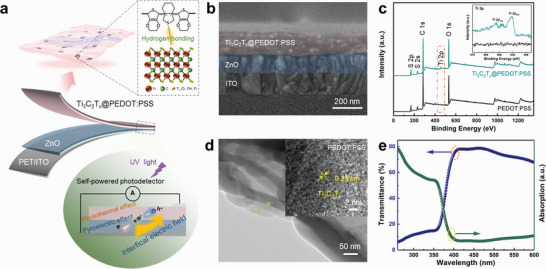
Device design and characterizations of the self‐powered photodetector. a) Schematic of device structure of the Ti_3_C_2_T*_x_* @PEDOT:PSS/ZnO heterojunction photodetector. b) Cross‐section SEM image of the Ti_3_C_2_T_x_@PEDOT:PSS/ZnO heterojunction photodetector. c) XPS survey scans spectra of Ti_3_C_2_T_x_@PEDOT:PSS nanohybrid and pristine PEDOT:PSS, the inset Ti 2p. d) TEM and HRTEM images of Ti_3_C_2_T_x_@PEDOT:PSS nanohybrid. e) Optical transmission and UV‐visible absorption spectra the Ti_3_C_2_T_x_@PEDOT:PSS/ZnO photodetector.

The strategy of designing a self‐powered UV photodetector with superior performance is depicted in **Figure** **1**a. Ti_3_C_2_T*_x_* MXene was employed to modify PEDOT:PSS, and then spin‐coated on ZnO film to construct organic/inorganic heterojunction with advantages of a simple fabrication process and flexibility. In view of the intrinsic pyroelectric effect of ZnO and compelling light‐to‐thermal conversion of Ti_3_C_2_T*_x_* MXene, we anticipate that an enhanced interfacial electric field can accelerate photo‐generated carrier separation. In addition, hydrogen bonding interaction deriving from –SO_3_H of PEDOT:PSS and –OH, –O, –F termination of Ti_3_C_2_T*_x_* screens the coulombic attraction between PEDOT and PSS chains, enabling PEDOT to transition into a liner structure, which is more favorable for charge transfer.^[^ ^15^ ^]^ Therefore, the Ti_3_C_2_T*_x_* @PEDOT:PSS/ZnO photodetector is constructed to boost charge utilization without external field for desirable practical application potential. The cross‐section scanning electron microscope (SEM) image of Ti_3_C_2_T_x_@PEDOT:PSS/ZnO heterojunction is presented in Figure 1b, from which the thickness of Ti_3_C_2_T_x_@PEDOT:PSS and ZnO films are measured to be 120 and 100 nm, respectively. The interface between Ti_3_C_2_T_x_@PEDOT:PSS and ZnO films can be clearly identified and the Ti_3_C_2_T*_x_* @PEDOT:PSS film is continuously and compactly coated on ZnO film. Initially, Ti_3_C_2_T*_x_* MXene was obtained by selectively etching the Ti_3_AlC_2_ MAX phase. The SEM image of multilayered Ti_3_C_2_T*_x_* showed a larger interlayer spacing after etching in Figure S1a,b (Supporting Information). To verify the formation of Ti_3_C_2_T*_x_* MXene, X‐ray diffraction measurement (XRD) was performed with results shown in Figure S1c (Supporting Information), which exhibits a characteristic peak (002) of Ti_3_C_2_T*_x_* at 2*θ* = 6.9. The delaminated Ti_3_C_2_T*_x_* nanosheet was fabricated by sonication‐assisted exfoliating of the multilayered Ti_3_C_2_T*_x_*. The thickness of Ti_3_C_2_T*_x_* nanosheet is extracted to be ≈1.6 nm and size of more than 1 µm based on the atomic force microscopy (AFM) image in Figure S2, Supporting Information. Subsequently, different content of Ti_3_C_2_T*_x_* nanosheets were introduced into PEDOT:PSS by a simple solution process to procedure Ti_3_C_2_T*_x_* @PEDOT:PSS nanohybrid. The XRD pattern in Figure S3 (Supporting Information) shows that peaks of the Ti_3_C_2_T*_x_* @PEDOT:PSS composite film confirmed the existence of two substances. Figure 1c demonstrates the X‐ray photoelectron spectroscopy (XPS) characterizations of PEDOT:PSS and Ti_3_C_2_T_x_@PEDOT:PSS films. In contrast to the pristine PEDOT:PSS without any Ti‐related peaks, Ti_3_C_2_T*_x_* @PEDOT:PSS shows the typical peaks of Ti 2p_3/2_ and Ti 2p_1/2_ at 455.3 and 461.2 eV (Insert in Figure 1c). Meanwhile, the high‐resolution C 1s XPS spectra of Ti_3_C_2_T*_x_* @PEDOT:PSS hybrid reveals four components corresponds to C–Ti–O (281.9 eV), C–C (284.3 eV), C–O (285.6 eV), and C≐O (290.1 eV) bonds, whereas of PEDOT:PSS shown three components (Figure S4, Supporting Information).^[^ ^16^ ^]^ These are corresponding to the high‐resolution Ti 2p and C 1s XPS spectra of Ti_3_C_2_T*_x_* MXene (Figure S5, Supporting Information). The morphology of Ti_3_C_2_T*_x_* @PEDOT:PSS was probed by transmission electron microscope (TEM) and the high‐resolution TEM (HRTEM) image further confirmed the hybrid structure (Figure 1d). The lattice fringes of Ti_3_C_2_T*_x_* with lattice spacing of ≈0.25 nm are clearly observed in Ti_3_C_2_T*_x_* @PEDOT:PSS hybrid structure. In order to quantitate photoactivity of the constructed device, the optical transmission and UV‐Vis absorption spectra of the Ti_3_C_2_T*_x_* @PEDOT:PSS/ZnO heterojunction are measured and displayed in Figure 1e. The optical absorption spectrum exhibits a sharp absorption edge at 380 nm, confirming the heterojunction's ability for absorption of UV light. The average transmittance of the device was >70% in the visible range, which demonstrated its suitability for transparent optoelectronics.^[^ ^17^ ^]^ Additionally, the transmission spectrum of the Ti_3_C_2_T*_x_* @PEDOT:PSS film was comparable to that of the pristine PEDOT:PSS film, which indicated that the modification of Ti_3_C_2_T*_x_* showed negligible effect on their film transmittance property (Figure S6a, Supporting Information). The high transmittance (above 90% from 250 to 800 nm) of Ti_3_C_2_T*_x_* @PEDOT:PSS film will have little effect on the solar‐blind region absorption of the photosensitive ZnO layer (Figure S6b, Supporting Information).^[^ ^18^ ^]^ All these results revealed that the Ti_3_C_2_T*_x_* @PEDOT:PSS/ZnO heterojunction can act as a promising candidate for UV sensing application due to its excellent selectivity in UV absorption and high transmittance in the visible region.

**Figure 2:**
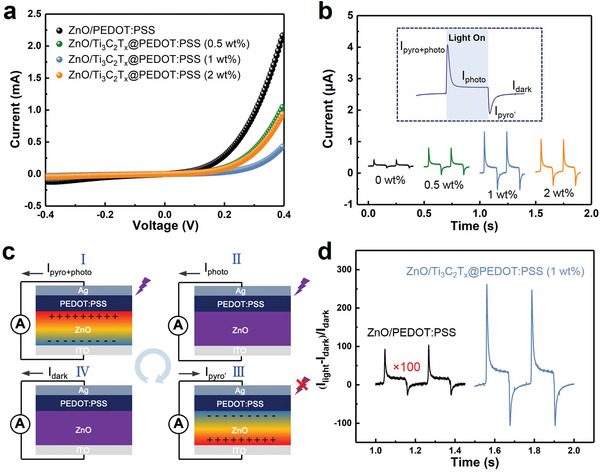
Photoresponse measurement of Ti_3_C_2_T*_x_* @PEDOT:PSS/ZnO (*x* wt.%) photodetectors. a) Typical *I–V* characteristics of PEDOT:PSS/ ZnO and Ti_3_C_2_T*_x_* @PEDOT:PSS/ZnO (0.5 wt.%, 1 wt.%, 2 wt.%) at dark condition. b) Time response curves of Ti_3_C_2_T*_x_* @PEDOT:PSS/ZnO (0, 0.5, 1, 2 wt.%) at 0 V under UV illumination. c) Schematic illustration of the working mechanism of the pyro‐phototronic effect corresponding to the four stages of photoresponse. d) On/off ratio of ZnO/PEDOT:PSS heterojunction photodetector without and with 1 wt.% Ti_3_C_2_T*_x_*.

The photoelectric performance of constructed PEDOT:PSS/ZnO photodetectors with different content of Ti_3_C_2_T*_x_* were tested. As shown in **Figure** **2**a, the current‐voltage (*I–V*) characteristics of Ti_3_C_2_T*_x_* @PEDOT:PSS/ZnO (0, 0.5, 1, 2 wt.%) photodetectors under dark conditions present typical photodiode behavior. Clearly, the threshold voltage appears to increase and then decrease with the increase of Ti_3_C_2_T*_x_* addition, indicating effective modulation of barrier height as well as charge carrier transport properties.^[^ ^7b^ ^]^ After that, the current‐time (*I–t*) characteristics of Ti_3_C_2_T_x_@PEDOT:PSS/ZnO heterojunction with different content of Ti_3_C_2_T*_x_* under UV illumination (355 nm, 1.9 mW) at zero bias were measured and illustrated in Figure 2b. Obviously, the detection performance of the self‐powered UV photodetector can be substantially modified by incorporating Ti_3_C_2_T*_x_* into PEDOT:PSS. The various concentration of added Ti_3_C_2_T*_x_* leads to different enhanced intensity for photocurrent under the same light intensity. All curves present an obvious four‐stage photocurrent dynamic behavior, which is caused by the pyro‐phototronic effect.^[^ ^7^, ^11^ ^]^ The working mechanism of the pyro‐phototronic effect is illustrated in Figure 2c. In the first stage, a spike photocurrent was induced by the combination of photovoltaic effect and pyroelectric effect on UV illumination which produced a fast temperature increase within ZnO. The corresponding photocurrent is labeled as *I*_pyro+photo_. With sustained light illumination, the temperature of ZnO film no longer changes and the pyroelectric effect vanishes. The photocurrent gradually decreases from *I*_pyro+photo_ to the plateau *I*_photo_ assigned to photovoltaic effect in the photodetector. When the UV light is turned off, the sudden decrease in temperature will cause the reverse distribution of pyroelectric charge across ZnO film resulting in the output current rapidly decreasing from *I*_photo_ to *I*_pyro’_. Lastly, with persistent dark conditions, the output current gradually recovers to *I*_dark._ Therefore, *I*_pyro+photo_ and *I*_photo_ are used to evaluate the photoresponse performances of the pyro‐phototronic effect‐based photodetectors. Evidently, compared to the PEDOT:PSS/ZnO sample, all of the Ti_3_C_2_T_x_@PEDOT:PSS/ZnO devices exhibited improved photocurrent. Ti_3_C_2_T*_x_* @PEDOT:PSS/ZnO (1 wt.%) (TPZ) photodetector presented the largest photocurrent among these heterojunction photodetectors illustrating the best separation and transfer of photo‐induced charge carriers. Photosensitivity is a key parameter to evaluate the performance of UV photodetectors, which can be defined as the ratio of photocurrent to dark current (*I*_on/off_) calculated as (*I*_light_‐*I*_dark_)/*I*_dark_. As shown in Figure 2d, the *I*_on/off_ of the TPZ photodetector is ≈260 times higher than pristine PEDOT:PSS/ZnO photodetector. As depicted in Figure S7a, Supporting Information, the dark current of the self‐powered TPZ photodetector is reduced by one order of magnitude compared to the pristine PEDOT:PSS/ZnO photodetector. In order to compare the response time of the PEDOT:PSS/ZnO and TPZ photodetectors, the time‐dependent current curves are normalized and shown in Figure S7b, Supporting Information. It is obvious that the rise time and the decay time of the TPZ photodetector is reduced by half than that of PEDOT:PSS/ZnO photodetector. Moreover, the I‐t characteristics of Ti_3_C_2_T*_x_* @PEDOT:PSS/ZnO heterojunction with different content of Ti_3_C_2_T*_x_* under 260 nm (0.92 µW) at zero bias were investigated and illustrated in Figure S8 (Supporting Information), where the Ti_3_C_2_T*_x_* @PEDOT:PSS/ZnO (1 wt.%) device also shows the highest performance. The above results unambiguously indicate that incorporating the Ti_3_C_2_T*_x_* nanosheets with an optimized concentration into PEDOT:PSS can significantly enhance photoresponse performance.

**Figure 3:**
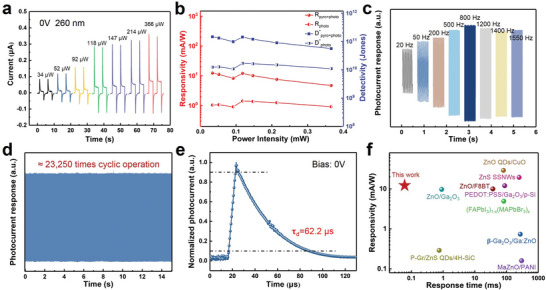
Comprehensive photoelectric properties of the self‐powered TPZ photodetector. a) *I–t* characteristics of the self‐powered TPZ photodetector under 260 nm illumination with different power intensity. b) *R*_pyro+photo_, *R*_photo_ and *D* ^*^_pyro+photo_, *D* ^*^_photo_ of the self‐powered TPZ photodetector as a function of power intensity. c) Time‐dependent photoresponse curve under different switching frequency illumination from 20 to 1550 Hz. d) Stability tests of the device, over 20 000 continuous response cycles of switch operations. e) Single period of plus response measured at 0 V bias. f) Comparison of R and response time for our device with previous reports on self‐powered solar‐blind photodetectors.

Thereafter, a comprehensive investigation of the important figures‐of‐merit of the TPZ photodetector was performed under 260 nm illumination at 0 V to represent its photodetection capability. The dynamic photoresponse of the self‐powered TPZ photodetector under various power intensities is illustrated in **Figure** **3**a. Obviously, *I*_pyro+photo_ and *I*_photo_ exhibit apparent intensity‐dependent characters, which corresponds well to the fact that the photo‐generation efficiency of charge carriers is proportional to the absorbed photo flux and larger temperature variations. To further evaluate the performance of the heterojunction, another two key parameters as responsivity and specific detectivity. It can be calculated as *R* = (*I*_light_ − *I*_dark_)/(*P_in_* S), where *I*_light_ is the photocurrent and *I*_dark_ is the dark current, *P* and *S* are the optical power density and the effective area of the photodetectors, respectively. The specific detectivity evaluate the ability of a device to detect weak light signals is given by $D^{*}=R/{\left(2eI_{\mathrm{dark}}/S\right)}^{1/2}$, where *R* is the responsivity and e is the elementary charge.^[^ ^19^ ^]^ Both D^*^ and R derived from the first stage with a combination of pyroelectric effect and photovoltaic effect (labeled as *D* ^*^_pyro+photo_ and *R*_pyro+photo_) present larger values than those obtained from the second stage (labeled as *D* ^*^_photo_ and *R*_photo_). As shown in Figure 3b, the maximum value of *R* is 12.3 and 1.04 mA W^−1^ for the first and second stages at the power intensity of 34 µW, respectively. Under the same condition, the maximum value of *D* ^*^ is observed as 1.47 × 10^11^ and 1.24 × 10^10^ Jones for two stages. Light switching frequency can significantly influence the pyro‐phototronic effect‐based photodetectors. Here the on/off cycles of the self‐powered TPZ photodetector toward UV illumination with chopper frequency increasing from 20 to 1550 Hz are demonstrated in Figure 3c. The reproducibility of photoresponse at high on‐off switching frequency was also investigated, which is essential for high‐performance photodetectors towards practical application. It can be observed that the photodetector exhibits a stable response over 23 250 cycles without degradation of performance (Figure 3d). The customized light source driving module has limited high frequency to ≈5 kHz while TPZ photodetector's stable and reversible photoresponse is still beyond 70.7% of the maximum value (3 dB bandwidth), indicating good sensitivity of the device for detecting the high‐speed flashing light signals. Correspondingly, the rise and fall time versus illumination frequency are extracted from Figure 3c and plotted in Figure S9 (Supporting Information). The response time decreases with increasing illumination frequency due to the enhanced temperature‐change rate under high frequency. The response speed describes the ability of photodetectors to track rapidly changing optical signals, which directly determines the speed of information transmission in the optical wireless communication system. In order to further explore the advantages of the TPZ photodetector in fast light detection, a pulse laser was used as the UV light source, and the photo‐generated electric signal was recorded in real time with an oscilloscope.^[^ ^14a^ ^]^ As demonstrated in Figure 3e, the single period of pulse response of the device at 0 V bias with a sharply rising and relatively slowly falling. Therefore, the decay time will determine the actual working bandwidth of the detector. The decay time (*τ*_d_, defined as the time required for the output signal changing from 90 to 10% of the peak output value) is ≈62.2 µs. The fast response speed of TPZ heterojunction photodetector is believed to be related to the quick separation and transportation of photo‐generated electron‐hole pairs by the internal electric field. These parameters are comparable to and even higher than most previously reported self‐powered solar‐blind photodetectors (Figure 3f and Table S1, Supporting Information).^[^ ^7^, ^9^, ^20^ ^]^ Furthermore, a bending test is also performed to explore the mechanical stability of the photodetector, which is crucial for practical application of a flexible photodetector. The device can operate well after different bending angles and the *I–t* curves are shown in Figure S10 (Supporting Information). Encouragingly, even though the device is bent at almost 160°, it still exhibits obvious photoresponse. The decreased photocurrent at this condition can be partly due to the reduced illumination area of the photodetector.

**Figure 4:**
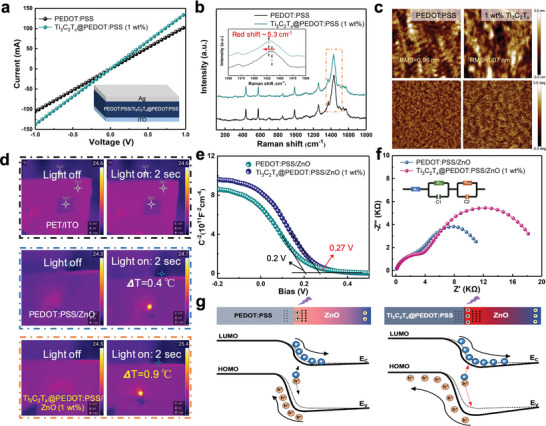
Mechanism analysis of Ti_3_C_2_T*_x_* modification enhanced photoresponse. a) I‐V dark curves of PEDOT:PSS and Ti_3_C_2_T_x_@PEDOT:PSS (1 wt.%) with a structure in the inset. b) Raman spectra of PEDOT:PSS and Ti_3_C_2_T_x_@PEDOT:PSS (1 wt.%). c) AFM topography height and phase images of pristine PEDOT:PSS and Ti_3_C_2_T_x_@PEDOT:PSS (1 wt.%) films. d) Thermal imaging of blank substrate, PEDOT:PSS/ZnO, and TPZ under UV illumination (2.14 mW). e) The typical Nyquist plots of control and TPZ photodetectors. The inset shows the equivalent circuit diagram. f) Mott‐Schottky plots of control and TPZ photodetectors. g) Band diagrams of PEDOT:PSS/ZnO and TPZ heterojunctions at the moment of light illumination.

To better understand the underlying mechanism of these improvements, a series of characterization analyses were carefully performed. As shown in **Figure** **4**a, the electrical conductivity properties of PEDOT:PSS and Ti_3_C_2_T*_x_* @PEDOT:PSS (1 wt.%) films were assessed from *I–V* measurement with the structure of the ITO/PEDOT:PSS with and without Ti_3_C_2_T*_x_* (1 wt.%)/Ag. The slopes of the curves are related to the conductivity of the films. Compared to the control PEDOT:PSS, the Ti_3_C_2_T*_x_* @PEDOT:PSS (1 wt.%) film exhibits higher slope values, which means the modified film has smaller contact resistance and higher hole mobility values.^[^ ^21^ ^]^ From the Raman spectroscopy in Figure 4b, pristine PEDOT:PSS exhibited a prominent peak at 1433 cm^−1^ corresponding to C*α* = C*β* symmetrical stretching vibration of the five‐membered thiophene rings on PEDOT. After the introduction of Ti_3_C_2_T*_x_*, this peak was red‐shifted. This shift can be ascribed to the introduction of PEDOT:PSS with Ti_3_C_2_T*_x_* incited the phase change of the PEDOT chains from benzoid to quinoid structure.^[^ ^15^, ^21^ ^]^ The quinoid conformation of PEDOT chains with stronger interchain interactions is favorable for the conductivity enhancement. The AFM characteristic is further employed to investigate the difference of the surface properties of the PEDOT:PSS and Ti_3_C_2_T_x_@PEDOT:PSS films. Figure 4c shows the topography height and phase images of a 1 × 1 µm^2^ area of the pristine PEDOT:PSS film and Ti_3_C_2_T*_x_* @PEDOT:PSS (1wt.%) film with the root‐mean‐square surface roughness of 0.97 and 1.06 nm, respectively. Obviously, the results demonstrated a favorable phase‐segregated morphology with the addition of Ti_3_C_2_T*_x_*, thus producing continuous interpenetrating conductive paths in thin film. However, introducing excess Ti_3_C_2_T*_x_* (2 wt.%) leads to an obviously aggregated morphology (Figure S11, Supporting Information), which could reduce the contribution to charge transfer and thus moderate performance improvement. Additionally, the XPS results of the pristine PEDOT:PSS film and Ti_3_C_2_T*_x_* @PEDOT:PSS (1 wt.%) film present in Figure S12 (Supporting Information), where the S 2p peaks between 162–166 eV and 166–172 eV binding energies that correspond to the sulfur atoms of PEDOT and PSS, respectively.^[^ ^22^ ^]^ The S 2p fitted signals at 163.3 eV and 164.5 eV are credited to the sulfur atoms of PEDOT, while the higher binding energies at 167.8 eV and 168.8 eV correspond to PSS. A chemical shift of S 2p at higher binding energy is observed upon incorporation of Ti_3_C_2_T*_x_*. The results demonstrated an evolution in the electronic environment of the sulfur atoms, which might be because of diminished coulombic interaction between PSS and PEDOT supporting the phase‐segregated results.^[^ ^15^, ^23^ ^]^ Therefore, the incorporation of Ti_3_C_2_T*_x_* enhances the charge transport kinetics of the PEDOT:PSS film, which is beneficial to prevent the charge recombination at the heterojunction interface. Moreover, a comparison of the absorption spectra of TPZ and PEDOT:PSS/ZnO heterojunctions shows that light utilization has a certain effect on performance improvement (Figure S13a, Supporting Information). An increased absorption in the UV region was noticed for TPZ heterojunction, which might be due to the UV light absorbance of Ti_3_C_2_T*_x_* MXene (Figure S13b, Supporting Information). Meanwhile, the thermal imaging of the substrate and devices under dark and 355 nm UV illumination are present in Figure 4d and Figure S14, Supporting Information. The TPZ device exhibits greater temperature variations, which can be explained by the high photothermal conversion efficiency of Ti_3_C_2_T*_x_* MXene.^[^ ^13c,d^ ^]^ The larger temperature change resulting in a stronger instantaneous pyro‐potential within ZnO further intensifies the pyro‐phototronic effect. The amount of transferred charges *Q*_pyro_ driven by the light‐induced pyroelectric effect in the external circuit is estimated by integrating the *I–t* curve above the reference value *I*_photo_.^[^ ^11^, ^12^ ^]^ The strong light‐self‐induced pyroelectric effect results in a large transferred charge *Q*_pyro_. Pyroelectric charges *Q*_pyro_ of TPZ and PEDOT:PSS/ZnO devices are calculated and investigated under various power intensities as shown in Figure S15, Supporting Information. Obviously, Ti_3_C_2_T*_x_* introducing can significantly improve the transferred charge *Q*_pyro_ representing the enhanced light‐induced pyroelectric effect. To further analyze the effect of Ti_3_C_2_T*_x_* introduction on the p‐n junction, the diode parameters such as ideality factor (*n*) and barrier height (*ϕ_b_*) of PEDOT:PSS/ZnO and TPZ devices are determined (Figure S16 and Note S1, Supporting Information). Compared to PEDOT:PSS/ZnO device, the *n* value of the TPZ device is slightly increased from 1.39 to 1.54 and the barrier height can also be calculated with a value increasing from 0.69 to 0.74 eV. Indeed, even a tiny change in the built‐in potential can have a significant effect on charge transport characteristics. The UV photoelectron spectroscopy (UPS) spectra and band gap of PEDOT:PSS and Ti_3_C_2_T_x_@PEDOT:PSS (1 wt.%) films were subsequently analyzed as shown in Figure S17 of Supporting Information. The E_HOMO_ is determined by *E*_HOMO_ = 21.22 *eV* − *E*_cutoff_ + HOMO, where *E*_cutoff_ is the energy at which the secondary photoemission begins and HOMO is the highest occupied molecular orbital position. The Ti_3_C_2_T*_x_* @PEDOT:PSS (1 wt.%) endows a higher HOMO level of 5.02 eV compared with the pristine PEDOT:PSS (5.18 eV), which broadens the space‐charge region and enhances the built‐in electric field.^[^ ^7^, ^24^ ^]^ Meanwhile, the built‐in potential (*V*_bi_) at PEDOT:PSS/ZnO and TPZ interface was estimated by Mott‐Schottky (M‐S) measurement under dark condition to confirm the built‐in electric field enhancement (Figure 4e). The *V*_bi_ of TPZ photodetector is larger than that of the control device, which means stronger extraction and separation efficiency of photo‐generated electron‐hole pairs at the interface, which is in agreement with the observations discussed above.^[^ ^25^ ^]^ On the other hand, electrochemical impedance spectroscopy (EIS) measurement is performed to explore the charge carriers transport and recombination dynamics in the devices. Figure 4f displays the Nyquist plots of PEDOT:PSS/ZnO and TPZ devices. The resistance values are fitted and extracted by the equivalent circuit model inserted in Figure 4f. *R*_s_, *R*_ct_, and *R*_rec_ denote series resistance, transport resistance, and recombination resistance, respectively. The fitted results of *R*_s_, *R*_ct_, and *R*_rec_ are listed in Table S2, Supporting Information. The decreased *R*_ct_ and obviously increased *R*_rec_ of the TPZ device indicates that the transfer of photo‐generated electron‐hole pairs was facilitated and their recombination was effectively suppressed. Figure 4g present the simulated energy band diagram of TPZ and PEDOT:PSS/ZnO heterojunctions. The introduction of Ti_3_C_2_T*_x_* generated transient thermal power facilitating change of temperature at the moment of UV illumination, which gave rise to enhanced positive pyroelectric charges at the ZnO side. Hence, more positive polarization charges at the TPZ heterojunction interface promote bending downward the energy band diagram resulting in the further increase of the barrier height of the heterojunction, which in turn effectively enhances separation and transportation of the photogenerated charge carriers.^[^ ^12b^ ^]^ Consequently, a series of complementary characterizations suggest that the synergy of improved light‐self‐induced pyroelectric polarization, increased built‐in electric field, and enhanced conductivity of PEDOT:PSS efficiently facilitated charge separation, accelerated charge transport, and suppressed interface charge recombination. The boosted charge utilization led to superior performance of the self‐powered TPZ photodetector.

**Figure 5:**
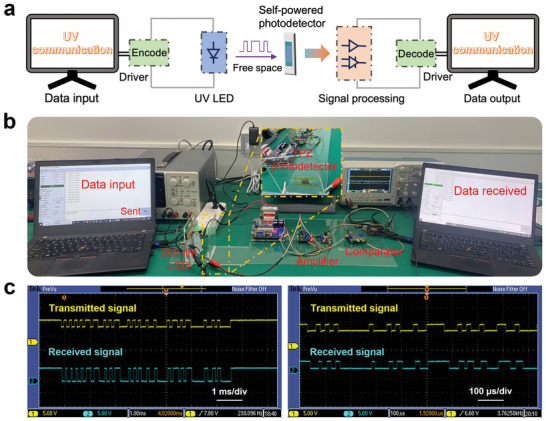
Demonstration of the self‐powered TPZ photodetector integrated into the UV communication system for efficient information transmission. a) Schematic illustration of the integrated UV communication system. b) The digital photograph of the UV communication with our TPZ photodetector as self‐powered optical signal receiver. c) Waveform comparison of the self‐powered TPZ photodetector with the message‐modulated light at different transmission rates.

UV communication with intrinsic advantages of anti‐interference and anti‐interception is highly appealing among optical wireless communication technologies. Self‐powered photodetectors as optical receivers are promising for improving portability and integration of UV communication systems. To verify the feasibility of practical application, the TPZ photodetector was integrated into a solar‐blind UV communication system as the self‐powered optical receiver to realize wireless information transmission. **Figure** **5**a shows the schematic diagram of the UV communication system designed here for data transmission demonstration, which includes data input/output devices, transmitter/receiver, amplifier, and comparator. Combining the 8‐bit encoding of the American Standard Code for Information Interchange (ASCII) with a high transmission rate meets the requirement for rapid complex information communication. The communication link used is the transmission of characters between computers. Relevant setting details are shown in Note S2 and Figure S18 of the Supporting Information. Through the integrated UV communication system, text and data files can be transmitted successfully, accurately, and rapidly. The processes for text transmittance can be illustrated as follows: First, the data flow from a computer was encrypted by ASCII and transferred into high and low voltage levels using a driver. Then, the modulated voltage level was used to high‐speed switch the UV light emitting diode (LED) with a light intensity of 340 µW to output a modulated light. The UV light is wirelessly transmitted in free space and received by the TPZ photodetector, which can convert the modulating light into the electric signal without external bias. After that, the output electric signals is processed by the amplifier and comparator and then input into driver, which can be decoded and transferred into data and displayed by another computer. The digital photograph of the UV communication system is present in Figure 5b. To test the applicability of real‐time high‐throughput information transmission, using UV light as information media, the input words “hello, USTB” and “UV Communication” were transmitted continuously and accurately under 9600 baud rate. Photographs of the user interface in the UV communication system were picked out and shown in Figure S19 of the Supporting Information. Besides, the input and output waveforms at different transmission rates are displayed in the oscilloscope (Figure 5c). The real‐time waveform comparison further clarifies that the self‐powered TPZ photodetector in the UV communication system can accurately and quickly restore the message‐modulated input waveforms. Additionally, the real‐time demonstration of the text transmission via the present UV communication system can be found in the videos (Video S1, Supporting Information).

## 3 Conclusion

In summary, we designed a self‐powered Ti_3_C_2_T*_x_* @PEDOT:PSS/ZnO heterojunction UV photodetector to boost charge utilization and enable desirable photoresponse performance. With a synergistic contribution of the photothermal effect intensifying pyro‐phototronic effect and the increased conductivity of the PEDOT:PSS, the device not only exhibits promoted charge separation and transport, but also suppressed interface charge recombination. The introduction of Ti_3_C_2_T*_x_* can efficiently reduce the dark current and response time, and promote the on/off ratio significantly compared to the pristine PEDOT:PSS/ZnO device. The optimized self‐powered TPZ photodetector delivers superior comprehensive performance with high responsivity of 12.3 mA W^−1^, fast response time of 62.2 µs together with stable cyclic operation. Notably, we successfully integrated the self‐powered TPZ photodetector into the UV communication system to present real‐time high‐throughput information transmission under 9600 baud rate. Our results suggest that high‐performance self‐powered photodetectors can potentially replace traditional high‐energy‐consuming UV detectors in OWC systems.

## 4 Experimental Section

### Preparation of Ti3C2T*_x_* MXene Colloidal Solution

Ti_3_C_2_T*_x_* MXene nanosheets were synthesized via the LiF/HCl selective etching process. 1 g of the Ti_3_AlC_2_ powders (400 mesh, Jilin 11 Technology Co., Ltd, China) was gradually added over 5 min into a 50 ml Teflon breaker containing 20 mL solution composed of 1 g LiF, 5 mL deionized H_2_O and 15 mL 12 M HCl (hydrochloric acid) under gentle stirring, as described in detail in previous reports. The reaction was allowed to continue for 24 h with stirring at 35 °C. Subsequently, the etched sample was washed with the deionized water at 3500 rpm for 6 min until the supernatant at around pH 6. Then multilayered Ti_3_C_2_T*_x_* was sonicated (80 W) in deionized water for 30 min to prepare the delaminated Ti_3_C_2_T*_x_*. Finally, single or few‐layer Ti_3_C_2_T*_x_* nanosheets were achieved by centrifugation for 30 min at 3500 rpm and the concentration of the prepared solution was ≈2 mg mL^−1^.

### Preparation of Ti_3_C_2_T_x_@PEDOT:PSS

Different volumes of Ti_3_C_2_T*_x_* nanosheets solution (5 µL, 10 µL, 20 µL) were mixed with 1 mL PEDOT:PSS solution to get Ti_3_C_2_T_x_ hybrid PEDOT:PSS solution with different concentrations (0.5 wt.%, 1 wt.%, 2 wt.% respectively). The hybrid solutions in vials were ultrasonicated for 20 min, ready for use.

### Device Fabrication

The PET/ITO flexible substrates with a sheet resistance of 8 Ω sq^−1^ were cleaned by sonication in isopropanol and ethanol subsequently for 10 min each, and dried by N_2_ flow. A ZnO film was deposited by radio frequency magnetron sputtering at a power of 80 W for 30 min. Then the pristine PEDOT:PSS and Ti_3_C_2_T*_x_* @PEDOT:PSS were spin‐coated on the surface of the ZnO film at 3000 rpm, followed by curing at 100 °C in ambient conditions for 10 min to remove the residual water. Then, a thin layer of silver was subsequently thermally evaporated on top of the ZnO/PEDOT:PSS and ZnO/Ti_3_C_2_T*_x_* @PEDOT:PSS heterostructures as the top electrode.

### Characterization and Measurements

TEM was measured on a FEI‐Tecnai G2 F20 TEM instrument. The morphologies and EDS were taken by FESEM (FEI QUANTA 3D FESEM). UV−vis absorption spectra was measured using a UV−vis−NIR spectrometer (Varian Cary 5000). XPS were measured by a Thermo Scientific K‐Alpha instrument. The KPFM and AFM measurements were taken on a commercially available AFM (Nanoscope IIID, Multimode). UPS was conducted with Helium lamp source emitting at 21.22 eV by Thermo Fisher Scientific ESCALab 250Xi system. EIS measurement was carried out on a CHI 660E electrochemical workstation with the frequency range from 10 Hz to 10^5^ Hz under zero bias in the dark condition. The Raman spectrum measurements were performed with confocal microscopy (JY‐HR800 and WITec CRM200) excitation under 532 nm laser. The typical photoresponse performance characteristics of the self‐powered PDs were measured and recorded by a semiconductor analysis system (Keithley 4200) and a low‐noise preamplifier (Stanford, SR570). The laser 355 nm laser with the spot size of 2 mm diameter and one single solar‐blind 260 nm UV LED were adopted as optical input stimuli. The light intensity was controlled by a continuously variable filter and measured by a power meter (Thorlabs PM 320E). An oscilloscope was used to visualize the transmitted signal. All tests were performed under ambient conditions at room temperature.

## Conflict of Interest

The authors declare no conflict of interest.

## Supporting information

Supporting Information

Supplemental Video 1

## Data Availability

The data that support the findings of this study are available from the corresponding author upon reasonable request.
