# Supplementary material for: Boosting Charge Utilization in Self‐Powered Photodetector for Real‐Time High‐Throughput Ultraviolet Communication
Source: Adv Sci (Weinh). 2023 Jun 4;10(23):2301585. doi: 10.1002/advs.202301585 (PMC10427366; doi:10.1002/advs.202301585)
Supplement: Supplementary file 1 — Supporting Information [file ADVS-10-2301585-s001.pdf]

ADVANCED SCIENCE
Open Access

Supporting Information

for *Adv. Sci.*, DOI 10.1002/advs.202301585

Boosting Charge Utilization in Self-Powered Photodetector for Real-Time High-Throughput Ultraviolet Communication

Tian Ouyang, Xuan Zhao, Xiaochen Xun, Fangfang Gao, Bin Zhao, Shuxin Bi, Qi Li, Qingliang Liao and Yue Zhang**

Supporting Information

Title: Boosting Charge Utilization in Self-powered Photodetector for Real-Time High-throughput Ultraviolet Communication

Tian Ouyang, Xuan Zhao, Xiaochen Xun, Fangfang Gao, Bin Zhao, Shuxin Bi, Qi Li, Qingliang Liao, Yue Zhang**

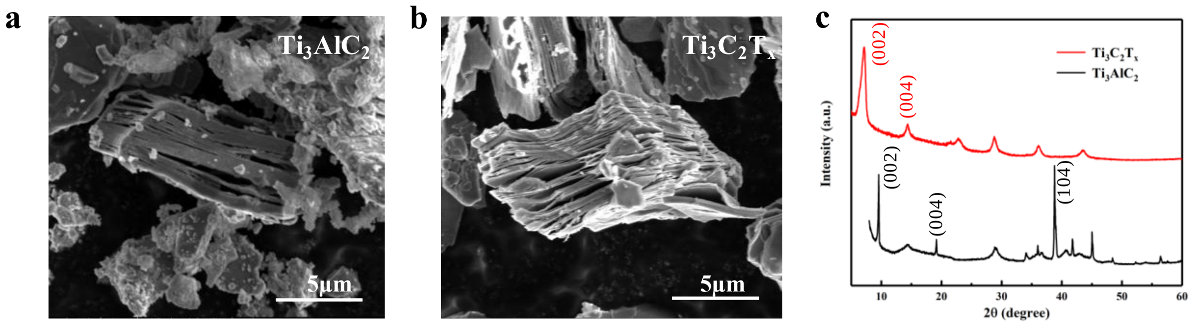

Figure S1. a, b) SEM images of Ti_3AlC_2 and $Ti_3C_2T_x$ power. c) XRD patterns of Ti_3AlC_2 and $Ti_3C_2T_x$.

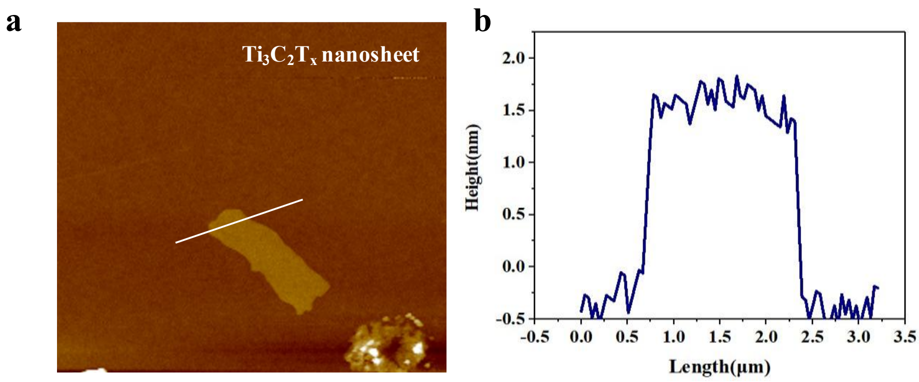

Figure S2. a) AFM image of single layer MXene nanosheet, and b) indicated the thickness of about 1.6 nm.

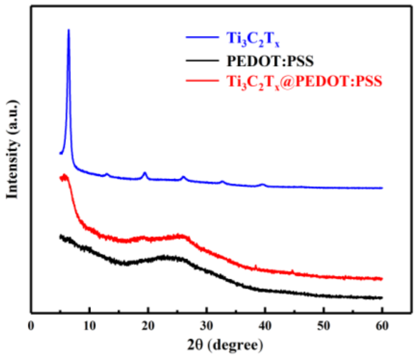

Figure S3. XRD patterns of $Ti_3C_2T_x$, PEDOT:PSS, and $Ti_3C_2T_x$@PEDOT:PSS.

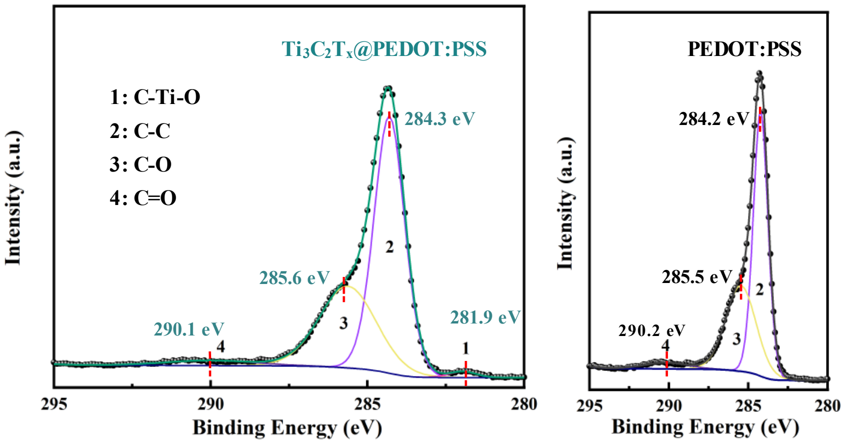

Figure S4. The high-resolution C 1s XPS spectra of PEDOT:PSS and $Ti_3C_2T_x$@PEDOT:PSS hybrid.

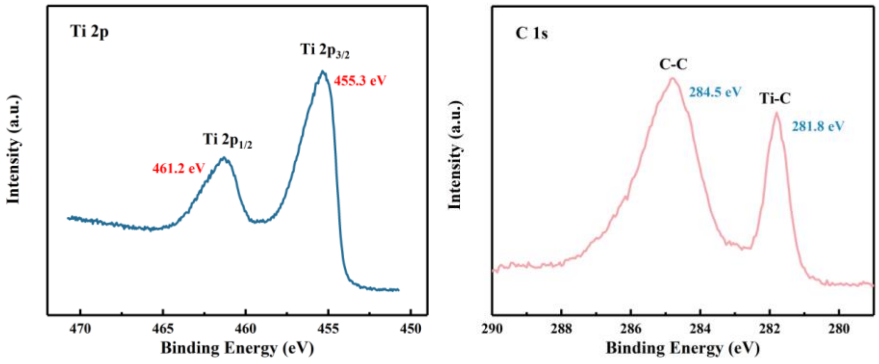

Figure S5. Ti 2p and C 1s XPS spectra of $Ti_3C_2T_x$ MXene.

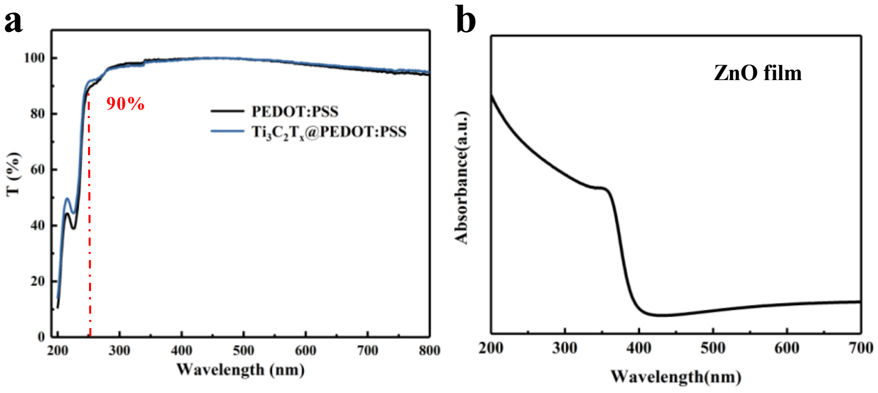

Figure S6. a) UV-vis absorption spectra of the ZnO film. b) Transmittance spectra of the PEDOT:PSS and $Ti_3C_2T_x$@PEDOT:PSS films.

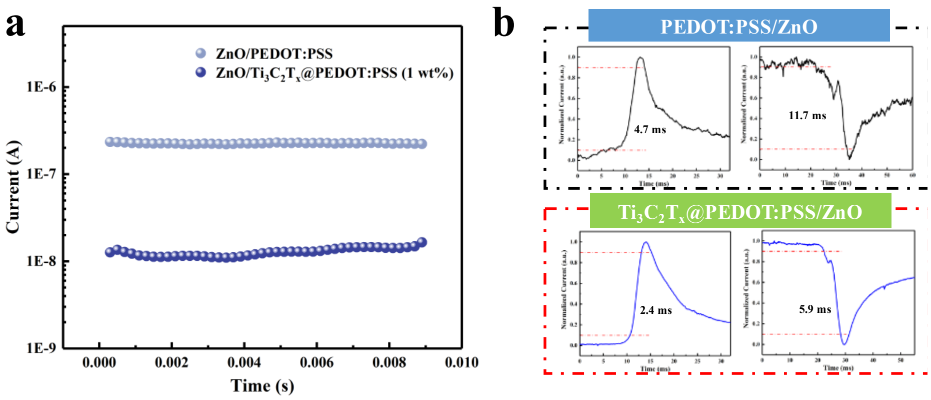

Figure S7. a) Dark current, b) Response time of PEDOT:PSS/ZnO and $Ti_3C_2T_x$@PEDOT:PSS/ZnO (1 wt%) photodetectors.

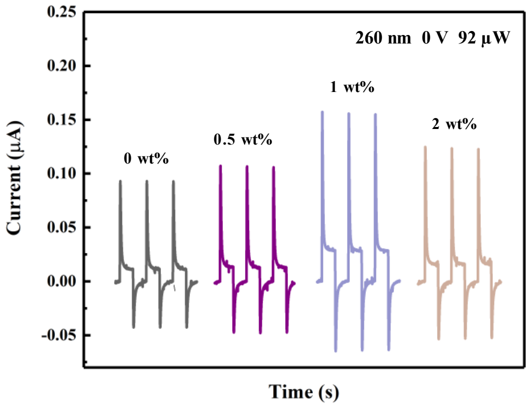

Figure S8. I-t curves of PEDOT:PSS/ZnO and $Ti_3C_2T_x$@PEDOT:PSS/ZnO (0.5 wt%, 1 wt%, 2 wt%) at 0V under 260 nm illumination.

Table S1. Comparison of the characteristic parameters of self-powered solar-blind UV photodetectors and the present work.

Device	Flexible/Transparent	Wavelength [nm]	Responsivity [mA/W]	Detectivity [Jones]	Response time [ms]	Reference
**MgZnO/PANI**	N/N	250	0.16	1.5×10^{11}	300/300	[20a]
**PEDOT:PSS/Ga_2O_3/p-Si**	N/N	255	12	-	60/88	[9b]
**ZnO/F8BT**	N/N	254	10	2.8×10^{11}	36.6/37	[7d]
**P-Gr/ZnS QDs/4H-SiC**	N/N	250	0.29	1.41×10^{10}	0.75/0.028	[20b]
**β-Ga_2O_3/Ga:ZnO**	N/N	254	0.736	-	179/272	[20c]
**ZnO/Ga_2O_3**	N/N	251	9.7	6.29×10^{12}	0.1/0.9	[20d]
**ZnO QDs/CuO**	N/N	244	29	6×10^{10}	80/80	[20e]
**$(FAPbI_3)_{1-x}(MAPbBr_3)_x$**	Y/N	254	4.92	7.57×10^{10}	82/64	[20f]
**ZnS SSNWs**	N/N	265	19.4	9.6×10^{11}	250/200	[20g]

**$Ti_3C_2T_x$@PEDOT:PSS/ ZnO**	Y/Y	260	12.3	1.47×10^{11}	0.02/0.062	**This work**

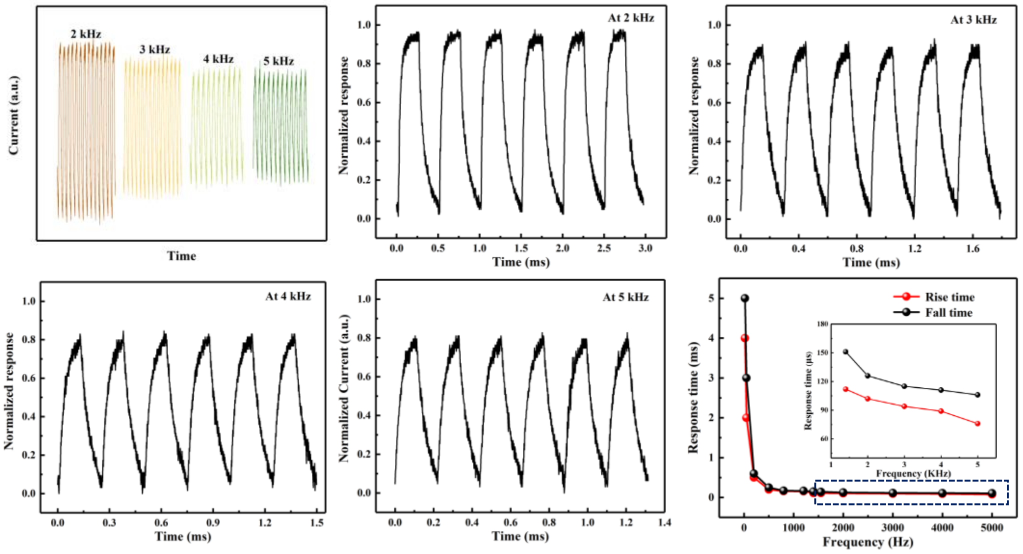

Figure S9. Light response at various frequencies.

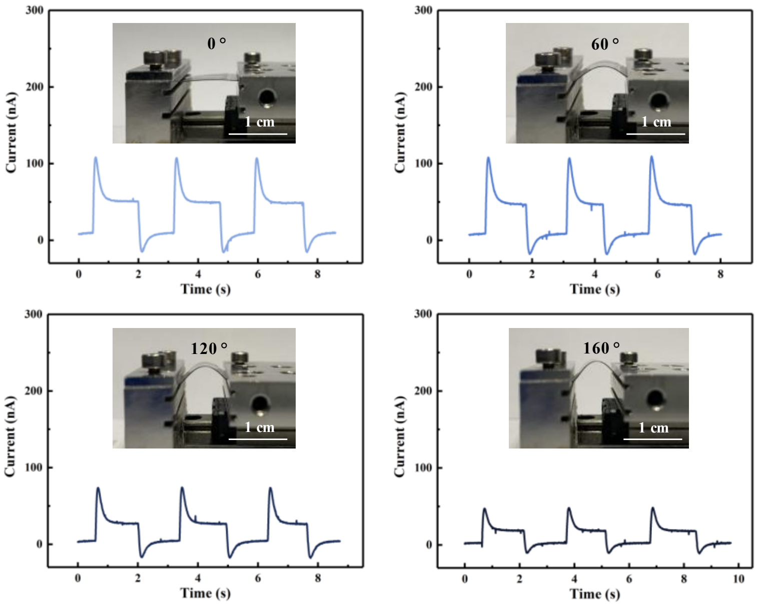

Figure S10. I-t curves of the self-powered TPZ device under UV light illumination after different bending angles.

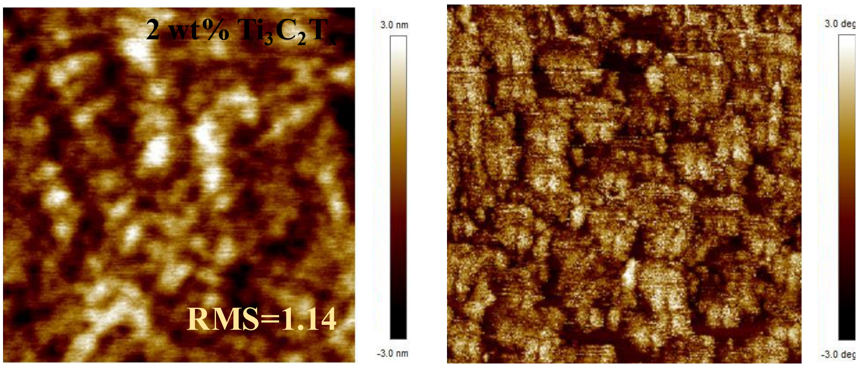

Figure S11. AFM height and phase images of $Ti_3C_2T_x$@PEDOT:PSS (2 wt%) film.

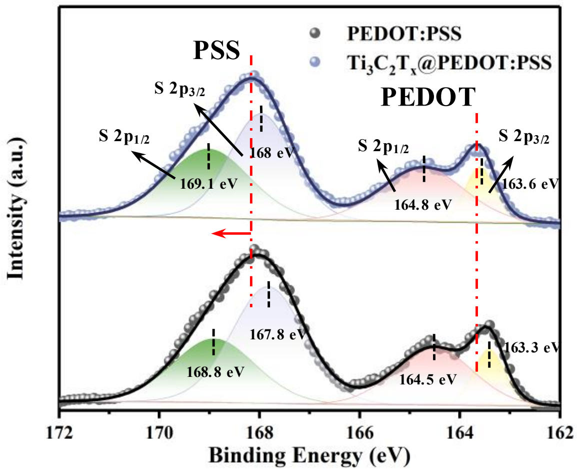

Figure S12. S 2p XPS spectra of pristine PEDOT:PSS and $Ti_3C_2T_x$@PEDOT:PSS (1 wt%) films.

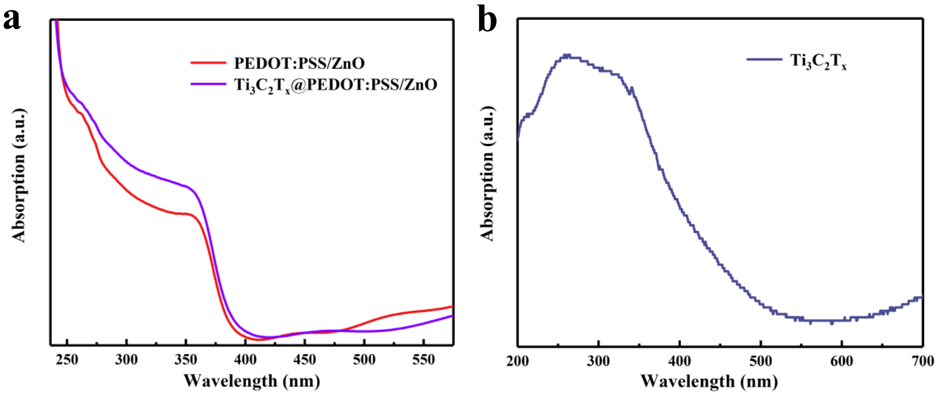

Figure S13. UV-vis absorption spectra of a) PEDOT:PSS/ZnO and $Ti_3C_2T_x$@PEDOT:PSS/ZnO heterojunctions b) $Ti_3C_2T_x$.

Table S2. Summary of EIS parameters of PEDOT:PSS/ZnO and $Ti_3C_2T_x$@PEDOT:PSS/ZnO devices.

Device	R_s (Ω)	R_{ct} (kΩ)	R_{rec} (kΩ)
PEDOT:PSS/ZnO	5.88	4.02	11.75

$Ti_3C_2T_x$@PEDOT:PSS/ZnO	17.66	3.68	16.93

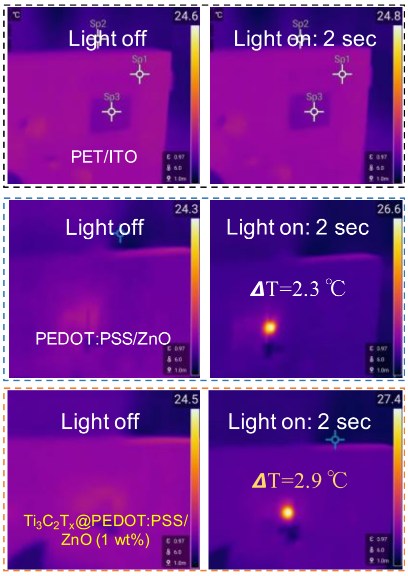

Figure S14. Thermal imaging of blank substrate, PEDOT:PSS/ZnO, and TPZ under UV illumination (4.08 mW).

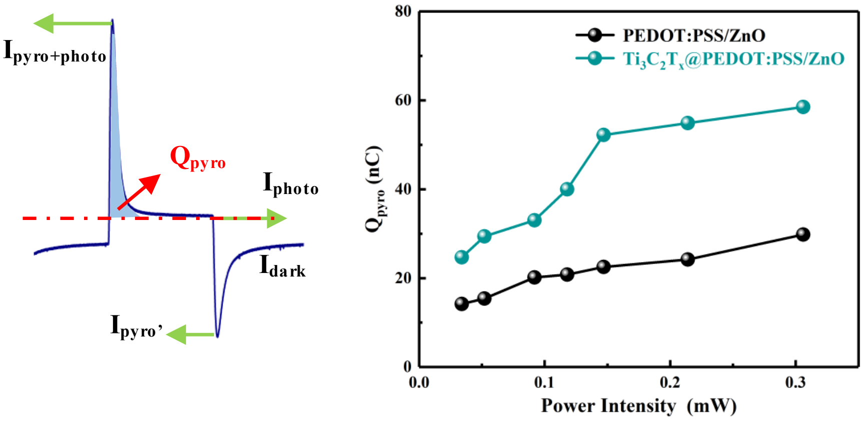

Figure S15. The equivalent pyroelectric charge of the PEDOT:PSS/ZnO and $Ti_3C_2T_x$@PEDOT:PSS/ZnO photodetectors as a function of power intensity.

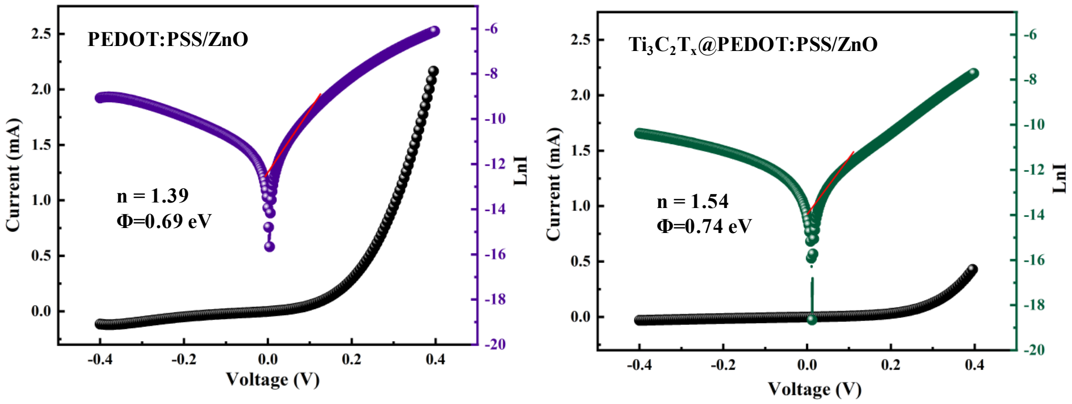

Figure S16. Typical I-V and LnI-V curves of the PEDOT:PSS/ZnO and $Ti_3C_2T_x$@PEDOT:PSS/ZnO devices.

Note S1: The I-V curves for PEDOT:PSS/ZnO device and $Ti_3C_2T_x$@PEDOT:PSS/ZnO device are fitted to extract the ideality factor n and barrier height ϕ_b. The diode behavior can be expressed as follows:

$$I = AA^*T^2 \exp\left(-\frac{q\phi_b}{kT}\right)\left[\exp\left(\frac{qV}{nkT}\right) - 1\right]$$

Where I and V represent current and voltage, respectively. A is the area, n is the ideality factor, k is the Boltzmann constant, q is the electronic charge, T is the Kelvin temperature and A^* is the effective Richardson constant and the A^* for ZnO is 32 A cm^{-2} K^{-2}.

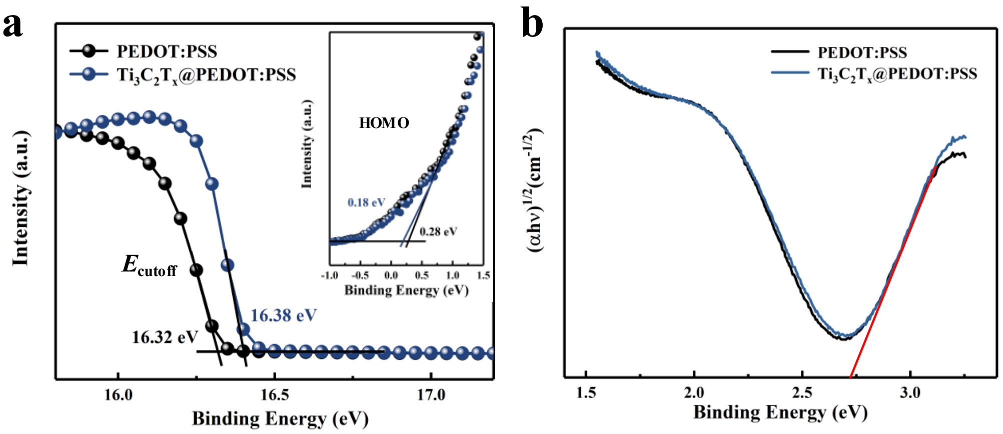

Figure S17. a) UPS spectrum of PEDOT:PSS films with $Ti_3C_2T_x$ of 0 wt% and 1 wt%. b) The bandgap of PEDOT:PSS films with $Ti_3C_2T_x$ of 0 wt% and 1 wt%.

Note S2: The sending and receiving computers pre-install serial debugger and set the parameters, including “Port Number”, “Baud rate”, “Data bits”, “Parity”, “Stop bits”. The user interface window of the serial program is shown in Figure S18. For two communication ports, these parameters must match. The serial communication parameters set by our UV communication system are: baud rate is 9600 Baud, data bit is 8 bits, parity bit is odd check, and stop bit is 1 bit. After completing the above steps, we test the wireless information transmission ability of the integrated UV communication system by inputting transmission characters in the serial debugger on the left computer side. The TPZ photodetector converts optical signals into electrical signals without external power supply, and after amplification and comparison is inputted into CH340G chip to realize signal demodulation, and then transmitted into the serial debugger presenting the received information on the right computer side. Therefore, the wireless communication link for the transmission of characters between computers has been completed.

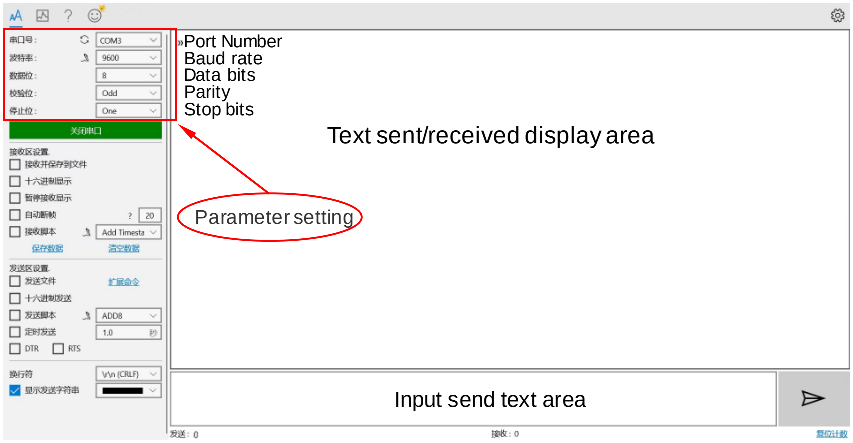

Figure S18. User interface window

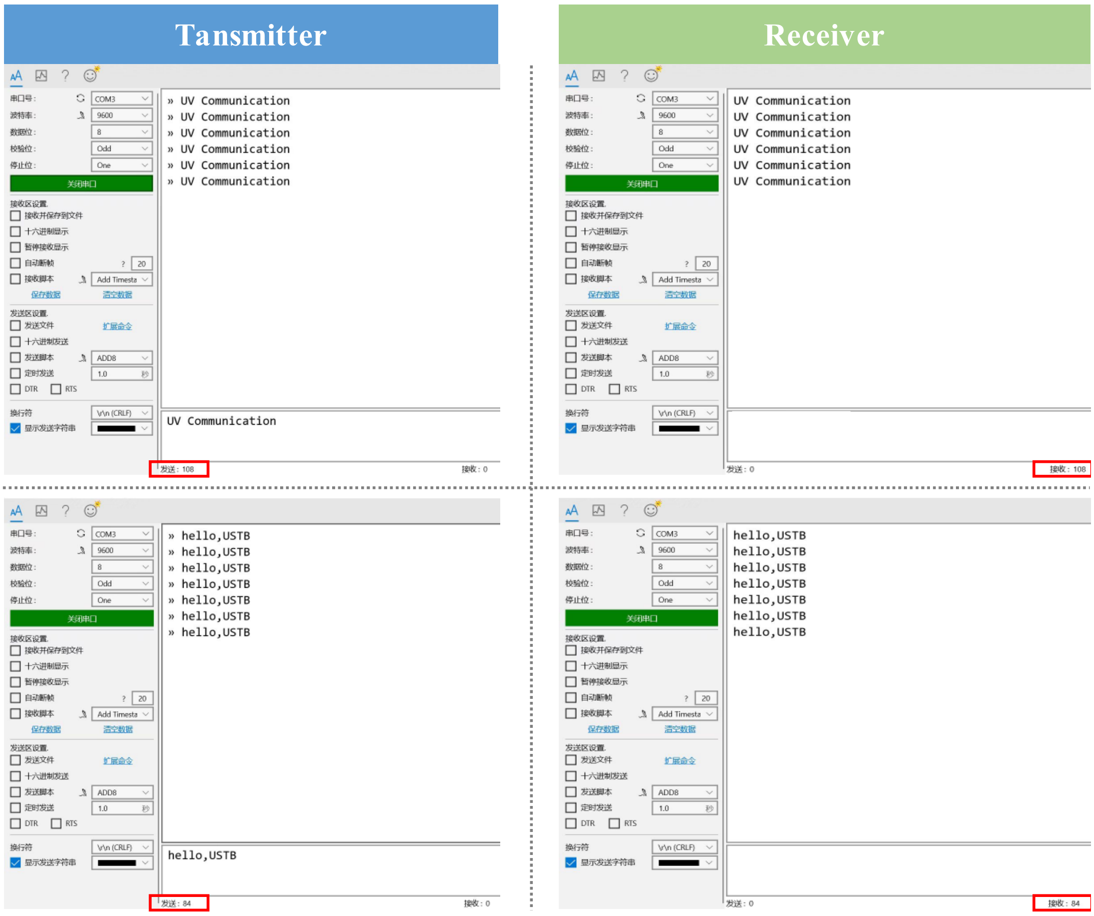

Figure S19. User interface of transmitter and receiver with transmission text of “hello, USTB” and “UV Communication”.
